# Investigation on two abnormal phenomena about thermal conductivity enhancement of BN/EG nanofluids

**DOI:** 10.1186/1556-276X-6-443

**Published:** 2011-07-09

**Authors:** Yanjiao Li, Jing'en Zhou, Zhifeng Luo, Simon Tung, Eric Schneider, Jiangtao Wu, Xiaojing Li

**Affiliations:** 1 State Key Laboratory for Mechanical Behavior of Materials, School of Materials Science and Engineering, Xi'an Jiaotong University, Xi'an, Shanxi, 710049, China; 2 Xi'an Research Inst. Of Hi-Tech, Hongqing Town, Xi'an, 710025, China; 3 GM R &D Center, 480-106-160, 30500 Mound Road Warren, MI 48090-9055, USA; 4 State Key Laboratory of Multiphase Flow in Power Engineering, Xi'an Jiaotong University, Xi'an, Shanxi 710049, China

## Abstract

The thermal conductivity of boron nitride/ethylene glycol (BN/EG) nanofluids was investigated by transient hot-wire method and two abnormal phenomena was reported. One is the abnormal higher thermal conductivity enhancement for BN/EG nanofluids at very low-volume fraction of particles, and the other is the thermal conductivity enhancement of BN/EG nanofluids synthesized with large BN nanoparticles (140 nm) which is higher than that synthesized with small BN nanoparticles (70 nm). The chain-like loose aggregation of nanoparticles is responsible for the abnormal increment of thermal conductivity enhancement for the BN/EG nanofluids at very low particles volume fraction. And the difference in specific surface area and aspect ratio of BN nanoparticles may be the main reasons for the abnormal difference between thermal conductivity enhancements for BN/EG nanofluids prepared with 140- and 70-nm BN nanoparticles, respectively.

## Introduction

The concept "nanofluids" was proposed by Choi [1] in 1995. Roughly speaking, nanofluids are solid-liquid composite materials consisting of solid nanoparticles or nanofibers with typically of 1-100 nm suspended in base liquid. Nanofluids provide a promising technical selection for enhancing heat transfer because of its anomalous high thermal conductivity and appear to be ideally suited for practical application with excellent stability and little or no penalty in pressure drop. As a result, nanofluids attract more and more interests theoretically and experimentally.

In the past decades, many investigations on thermal conductivity enhancement of nanofluids have been reported. These papers mainly focused on factors influencing thermal conductivity enhancement [2-16], mechanism for thermal conductivity enhancement [17-22], model for predicting the enhancement of thermal conductivity [23-29]. Recently, controversy about whether the dramatic increase of thermal conductivity with small nanoparticle loading in nanofluids is true was reported [30,31]. Some researches showed that no anomalous enhancement of thermal conductivity with small nanoparticle loading was achieved in the nanofluids and the thermal conductivity enhancement is moderate and can be predicted by effective medium theories. Besides, the mechanism of thermal conductivity enhancement is a hotly debated topic now, and many researchers pay attention to the influence of aggregation, morphology, and size of nanoparticles on thermal conductivity enhancement of nanofluids [32-39].

Focus on the current research interest, boron nitride/ethylene glycol (BN/EG) nanofluid was synthesized by a two-step method. The effect of particles volume fraction and size of nanoparticles on thermal conductivity enhancement were investigated and two abnormal phenomena were observed. In present paper, the two abnormal phenomena are reported and the mechanism of thermal conductivity enhancement is discussed.

## Experimental

**Figure 1:**
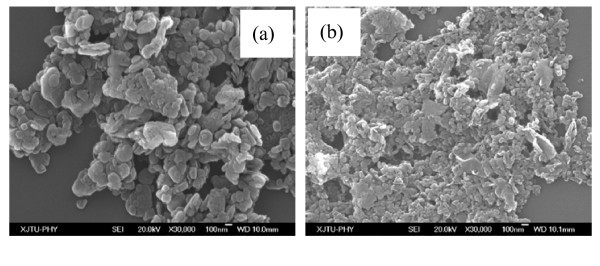
**SEM image of the BN nanoparticles**. (a) 140nm (b) 70nm.

BN powder of 140 and 70 nm with purity more than 99% were used as additives, as shown in Figure 1a, b, and ethylene glycol in analytical grade was employed as basefluid to prepare BN/EG nanofluids. A two-step method was used to synthesize BN/EG nanofluids. Proper quantities of BN powder weighed by a mass balance with an accuracy of 0.1 mg were dispersed into the ethylene alcohol base fluid. No dispersant was added. In order to assure uniform dispersion of nanoparticles in the base fluid, magnetic force stirring and ultrasonic agitation for 30 min were then employed, respectively. The apparatus and parameters for preparing nanofluids are shown in Table 1. The morphology of the dry nanoparticles was observed by a JEOL JSM-7000F scanning electron microscope (JEOL Ltd., Tokyo, Japan) and the nanoparticles suspended in the nanofluid were observed by a JEM-200CX transmission electron microscope (TEM; JEOL Ltd). The specific surface area of the nanosized BN powders were measured by Brunnauer-Emmett-Teller methods using a micromeritics ASAP 2020 surface area and porosity analyzer (Micromeritics Instrument Corp., Norcross, GA, USA). The Crystalline structure of the BN nanoparticles was investigated by means of Rigaku D/MAX-2400 x-ray diffraction analysis (Rigaku Corp., Tokyo, Japan) (XRD) using Cu Ka radiation (λ = 0.15418nm) at room temperature. The thermal conductivity of the BN/EG nanofluids was measured by transient hot-wire apparatus [40]. The uncertainty of this apparatus is between ± 2.0%. To improve the accuracy of the data, the thermal conductivity of BN/EG nanofluids with lower nanoparticles volume fraction was measured by an improved transient hot-wire apparatus [41]. This improved transient hot-wire apparatus is simpler and more robust compared to previous ones besides the improvement on accuracy [42,43]. The uncertainty of the improved transient hot-wire apparatus is between ± 0.51%.

**Table 1:** Apparatus for preparing nanofluids

Apparatus	Specification	Power	Revolution speed/frequency
Magnetic force stirring	78HW-1	25 W	1,600 rpm
Ultrasonic agitation	SK1200H	45 W	59 Hz

## Results and discussion

To investigate the effect of nanoparticle volume fraction on thermal conductivity enhancement of BN/EG nanofluids, 0.2, 0.6, 1.0, 2.0, 3.0, 4.0, and 5.5 vol.% BN/EG nanofluids were synthesized and thermal conductivity of them was measured. The average size of the BN nanoparticles used in these nanofluids is 140 nm. BN/EG nanofluid (1.25 vol.%) with the BN nanoparticles of 140 nm was prepared and its thermal conductivity was measured after depositing for 216 days. The volume fraction of the 1.25 vol.% BN/EG nanofluids after the depositing was 0.025 vol.%. In order to examine the effect of nanoparticle size on thermal conductivity enhancement, 70-nm BN nanoparticles were used as additives to synthesize BN/EG nanofluids with the nanoparticles volume fraction of 1.0% to 5.5% and thermal conductivity of them was measured. The thermal conductivity enhancement of these nanofluids was calculated, as shown in Table 2. The measured thermal conductivity of ethylene alcohol was 0.247 W/mK. The data marked with an asterisk (*) was measured by an improved transient hot-wire apparatus [41].

**Table 2:** Thermal conductivity enhancement of the BN/EG nanofluids

Volume fraction (vol.%)		0.025	0.2	0.6	1.0	2.0	3.0	4.0	5.5
Δ*k*/*k *(%)	140 nm	2.0*	0.8*	3.2*	5.7*	10.8	14.9	20.3	30.3
	70 nm	-	-	-	4.5	7.2	11.8	18.3	24.5

**Figure 2:**
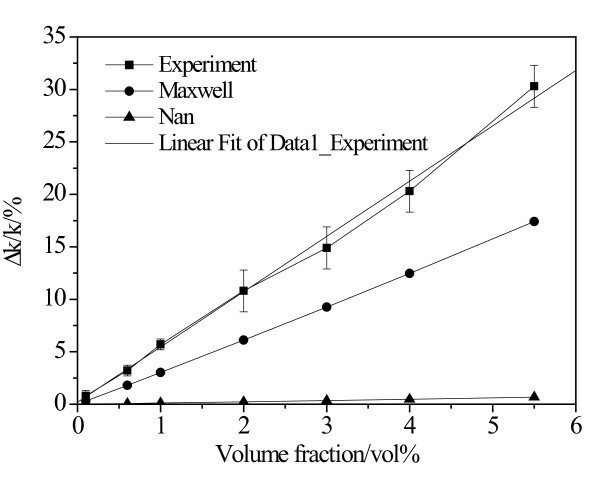
**Comparison of experimental results and theoretical model on thermal conductivity enhancement of BN/EG nanofluids vs. volume fraction of BN nanoparticles**.

Figure 2 shows the thermal conductivity enhancement of BN/EG nanofluids as a function of particle volume fraction. For volume fraction varying from 0.2 vol.% to 5.5 vol.%, data fitting indicates that the thermal conductivity enhancement of BN/EG nanofluids increases linearly with the increment of nanoparticle volume fraction. The *R *value is 0.9981. The thermal conductivity enhancement predicted by Maxwell's model [44] and Nan's model [27] were also illustrated in Figure 2. Based on Maxwell's work, the effective thermal conductivity of a homogeneous suspension can be predicted as (Maxwell, 1873)(1)

where *k_p _*is the thermal conductivity of the dispersed particles, *k_f _*is the thermal conductivity of the dispersion liquid, and *ϕ *is the particle volume concentration of the suspension.

Equation 1 is valid for well-dispersed non-interacting spherical particles with negligible thermal resistance at the particle/fluid interface. Considering the effects of particle geometry and finite interfacial resistance, Nan et al. generalized Maxwell's model to yield the following expression for the thermal conductivity ratio:(2)

where for particles shaped as prolate ellipsoids with principal axes *a*_11 _= *a*_22 _>*a*_33_

α_11_, α_22_ and α_33_  are, respectively, radii of the ellipsoid along the ,   and  axes of this ellipsoidal composite unit cell, *L_ii_* are well-known geometrical factors dependent on the particle shape, *p* is the aspect ratio of the ellipsoide, *k_m_* is the thermal conductivity of the matrix phase,   is quivalent thermal conductivities along the  symmetric axis of this ellipsoidal composite unit cell, and *R_bd _*is the Kapitza interfacial thermal resistance.

The conventional Maxwell model and Nan's model severely underestimates the enhancement of thermal conductivity for BN/EG nanofluids. It may be ascribed to that Maxwell model only takes the effect of particle volume fraction into account for thermal conductivity enhancement of nanofluids without considering the effect of particle shape, nanolayers at solid/liquid interface, and Brownian motion of nanoparticles and others. Nan's model is for particulate composites not for nanofluids. Although Nan's model considered the effect of nanoparticle shape and finite interfacial resistance, the effects of Brownian motion and aggregation of nanoparticles on thermal conductivity of nanofluids cannot be ignored. Now, no suitable model proposed by other researchers can fit well with the data we got. It is necessary to develop a new model considering all important factors influencing the thermal conductivity enhancement of BN/EG nanofluids. The work about this issue is being done by our group and will be reported later.

**Figure 3:**
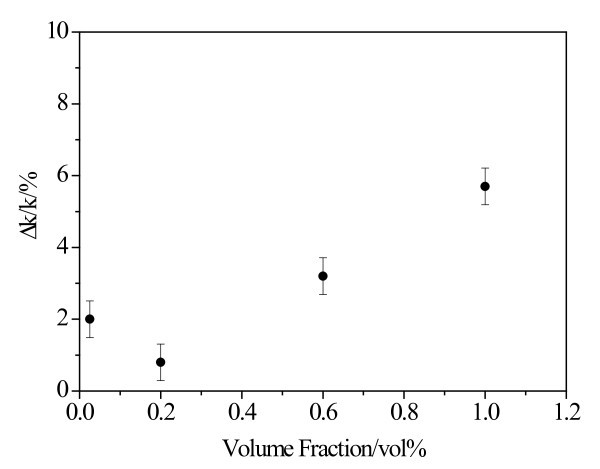
**Thermal conductivity enhancement of BN/EG nanofluids**.

Some investigations reported in literature indicate that thermal conductivity enhancement will increase with the increment of volume fraction of nanoparticle [4,9,10]. That is to say that the thermal conductivity enhancement for nanofluids with low nanoparticle volume fraction must be lower than that of nanofluids with high-volume fraction of nanoparticle. But an absolutely different phenomenon was observed in current experiment. A 2.0% enhancement of thermal conductivity was obtained for 0.025 vol.% BN/EG nanofluids prepared by setting 1.25 vol.% BN (140 nm)/EG nanofluids for 216 days, which is much higher than a 0.8% increment for 0.2 vol.% BN (140 nm)/EG nanofluids prepared by a two-step method, as shown in Figure 3. To find the reason for this abnormal thermal conductivity enhancement, high-resolution TEM (HRTEM) observation of the nanoparticles suspended in the nanofluids was conducted.

**Figure 4:**
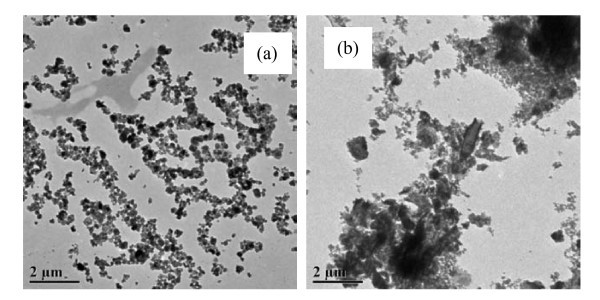
**HRTEM micrographs of BN nanoparticles suspended in BN/EG nanofluids**. (a) Chain-like loose aggregation of BN nanoparticles in 0.025vol% BN/EG nanofluids. (b) Cloud-like compact aggregation of BN nanoparticles in 0.2Vol% BN/EG nanofluids.

Figure 4a, b showed the morphology of nanoparticles suspended in 0.025 vol.% and 0.2 vol.% BN/EG nanofluids, respectively. It can be seen that the morphology of BN nanoparticles suspended in 0.025 vol.% BN/EG nanofluids is chain-like loose aggregation while in 0.2 vol.% BN/EG nanofluids is cloud-like compact aggregation. This discrepancy may be the main reason for the abnormal difference between the thermal conductivity enhancements of them. Generally, Brownian motion, by which particles move through liquid, thereby enabling direct solid-solid transport of heat from one to another, is considered as a key mechanism governing the thermal behavior of nanofluids [17-20]. In 0.025 vol.% BN/EG nanofluids, many uniform distributed chain-like loose aggregations of nanoparticles, acting as a three-dimensional dense network, can improve the heat transfer efficiency by providing many rapid, longer heat flow paths through Brownian motion of nanoparticles. While in 0.2 vol.% BN/EG nanofluids, high efficient heat transfer was limited in cloud-like compact aggregation. Heat transfer among cloud-like compact aggregations would be weakened for the large regions of particle-free liquid with high thermal resistance. It can be speculated that a more high thermal conductivity could be obtained when the volume fraction of these uniform distributed chain-like loose aggregations of nanoparticles in BN/EG nanofluid was increased because more efficient heat flow paths in the nanofluid could be provided.

The volume fraction of this 0.025 vol.% BN/EG nanofluids was measured after sedimentation for 120 days and the value of it is 0.017 vol.%. This phenomenon indicated that the stability of the nanofluid is excellent. And the long-term stability of this nanofluid may be ascribed to the flake-like morphology and incompact aggregation of the BN nanoparticles, as showed in Figure 4a. It can be expected that the stability of this nanofluid can be improved further when some appropriate dispersant was used. The phenomenon mentioned above indicates that nanofluids with high thermal conductivity and long-term stability can be obtained by adding relatively lower volume fraction of nanoparticles when the nanoparticles suspended in base liquid with proper morphology and aggregation. This kind of nanofluid is promising for engineering application.

**Figure 5:**
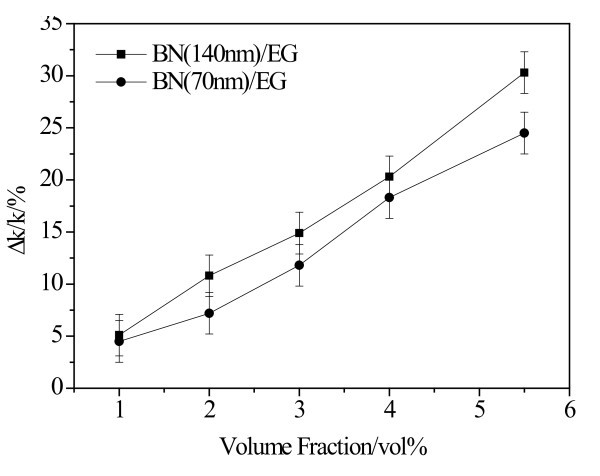
**Thermal conductivity enhancement vs. volume fraction for BN/EG nanofluids with different size of BN nanoparticles**.

Size of nanoparticles is an important factor influencing thermal conductivity of nanofluids because shrinking it down to nanoscale not only increases the surface area relative to volume but also generates some nanoscale mechanisms in the suspensions [18,24]. Theoretical evidence [18,24,35] indicate that the effective thermal conductivity of nanofluids increases with decreasing particle size. Some experimental research [36-39] showed that as the nanoparticle diameter is reduced, the effective thermal conductivity of nanofluids becomes larger. The reason for this phenomenon was interpreted as the high specific surface area of small nanoparticles and intensified micro-convection provoked by small nanoparticles. While in present study, thermal conductivities of BN/EG nanofluids synthesized with 140- and 70-nm BN nanoparticles were measured and a different phenomenon was observed, as shown in Figure 5. It can be found that the thermal conductivity enhancement of nanofluids synthesized with large size (140 nm) BN nanoparticles is higher than that synthesized with small size (70 nm) BN nanoparticles. Hong [4] and Xie [9] also found similar phenomenon. This phenomenon is much different from the normal rule. What was the reason for this abnormal difference in thermal conductivity enhancement? The author believed that it can be ascribed to the difference in shape of the BN nanoparticles by x-ray diffraction (XRD) analysis, HRTEM images, and specific surface area of the BN nanoparticles.

**Figure 6:**
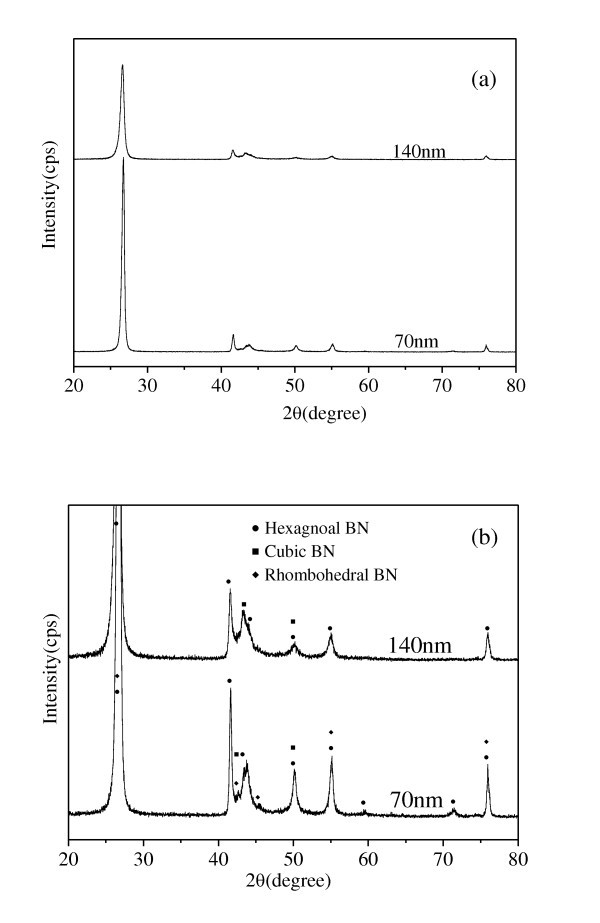
**XRD patterns of the BN nanoparticles**. (a) Original pattern (b) partial enlarged pattern.

Figure 6a is an XRD pattern of the BN powder with different size. Figure 6b is a partial enlarged pattern of Figure 6a. From Figure 6b, we can observe that the BN powder was composed of different phases. Hexagonal BN and cubic BN are the main phases for 140-nm BN powder. The weight ratio of hexagonal BN and cubic BN is about 93:7 through qualitative analysis made by the software attached by the Rigaku D/MAX-2400 x-ray diffraction analysis. For 70-nm BN powder, hexagonal BN, rhombohedral BN, and cubic BN are the main phases and the weight ratio of these three different phases is about 62:35:3. So we can conclude that the 140-nm BN powder are mainly composed of flake-like hexagonal BN while 70-nm BN powder are composed of 62% flake-like hexagonal BN and 38% BN with different shape.

**Figure 7:**
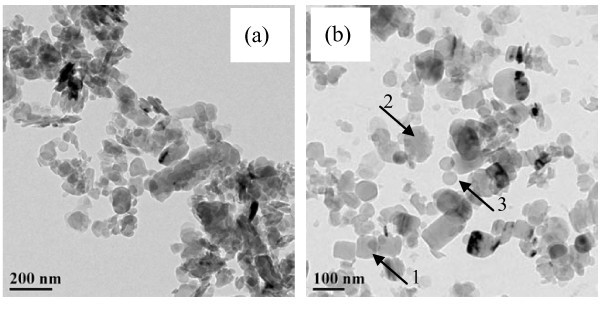
**HRTEM image of the BN nanoparticles**. (a) 140 nm (b) 70 nm.

Further observation on HRTEM image of these two kinds of BN nanoparticles indicates that the qualitative analysis about the phase component of 140-nm BN nanoparticles and 70-nm BN nanoparticles is correct, as shown in Figure 7a, b. The morphology of 140-nm BN nanoparticles is nearly all ellipsoid. However, the morphology of 70-nm BN nanoparticles is composed of cubic, ellipsoid, and spherical shape, as marked by arrows 1, 2, and 3 in Figure 7b. Moreover, the shape for most 70-nm BN nanoparticles is cubic and spherical, only few ellipsoid nanoparticles are observed. This difference in morphology indicate that nearly all 140-nm BN nanoparticles is composed of flake-like H-BN nanoparticles while 70-nm BN nanoparticles is composed of fewer flake-like H-BN and many cubic and spherical BN nanoparticles. For the specific surface area of flake-like nanoparticles is higher than that of cubic and spherical nanoparticles, the specific surface area of 140-nm BN nanoparticles is expected to be higher than that of 70-nm BN nanoparticles. Experiment showed that the specific surface area of 140-nm BN powder is 40.6098 m^2^/g while that of 70-nm BN powder is 35.71 m^2^/g. Besides, the aspect ratio of ellipsoid nanoparticles is higher than that of cubic and spherical nanoparticles. So the aspect ratio of 140-nm BN nanoparticles is higher than that of 70-nm BN nanoparticles. These differences in specific surface area and aspect ratio of 140-nm BN nanoparticles and 70-nm BN nanoparticles may be the main reasons for the abnormal different in thermal conductivity enhancement because heat transfer between the nanoparticle and the base fluid can be promoted for the larger specific surface area. Furthermore, rapid, longer heat flow paths are apt to the formation between higher aspect ratio nanoparticles and these heat flow paths can promote heat transfer also. The action of these two aspects leads to the enhancement of thermal conductivity for BN/EG nanofluids synthesis with 140-nm BN nanoparticles is higher than that synthesized with 70-nm BN nanoparticles.

## Conclusions

In summary, two abnormal phenomena about thermal conductivity enhancement of BN/EG nanofluids was investigated. One is the abnormal increment of thermal conductivity for BN/EG nanofluids at very low volume fraction, and the other is the abnormal thermal conductivity enhancement for BN/EG nanofluids synthesized with different size of BN nanoparticles. The chain-like loose aggregation of nanoparticles is responsible for the abnormal increment of thermal conductivity in the BN/EG nanofluids with very low particles volume fraction. And the difference in specific surface area and aspect ratio of BN nanoparticles may be the main reason for the abnormal difference between thermal conductivity enhancements for BN/EG nanofluids prepared with 140 and 70-nm BN nanoparticles, respectively.

## Competing interests

The authors declare that they have no competing interests.

## Authors' contributions

YL carried out the experimental studies and drafted the manuscript. JZ guide the experimental studies and revised the manuscript. ZL carried out part of the experimental studies. ST and ES participated in experiment design and coordination. JW, XL participated in the measurement of part of the thermal conductivity data. All authors read and approved the final manuscript.
